# Quantification of vendor-specific relationships between fast gradient echo and steady-state free precession cine MRI for determination of myocardial mass and volumes

**DOI:** 10.1186/1532-429X-11-S1-P240

**Published:** 2009-01-28

**Authors:** Andrew S Chi, W Craig Johnson, Elzbieta Chamera, John Eng, Joan Bathon, Chia-Ying Liu, Joao AC Lima, David A Bluemke

**Affiliations:** 1 Johns Hopkins, Baltimore, MD USA; 2 University of Washington, Seattle, WA USA

**Keywords:** Left Ventricular Mass, Interobserver Variability, Cardiac Mass, Fast Gradient, Cine Sequence

## Objectives

To quantify the vendor-specific relationships between fast gradient echo and steady-state free precession for determination of cardiac mass and volumes.

## Background

Steady-state free precession (SSFP) cine sequences have replaced fast gradient echo (FGRE) cine given the advantages of SSFP to provide faster image acquisition, increased contrast to noise ratio, increased blood-tissue contrast, and decreased interobserver variability as compared to FGRE. Substantial differences between SSFP and FGRE for quantification of cardiac mass and volumes have been reported, but differences between vendor results using both pulse sequences has not been characterized.

## Methods

**Figure 1:**
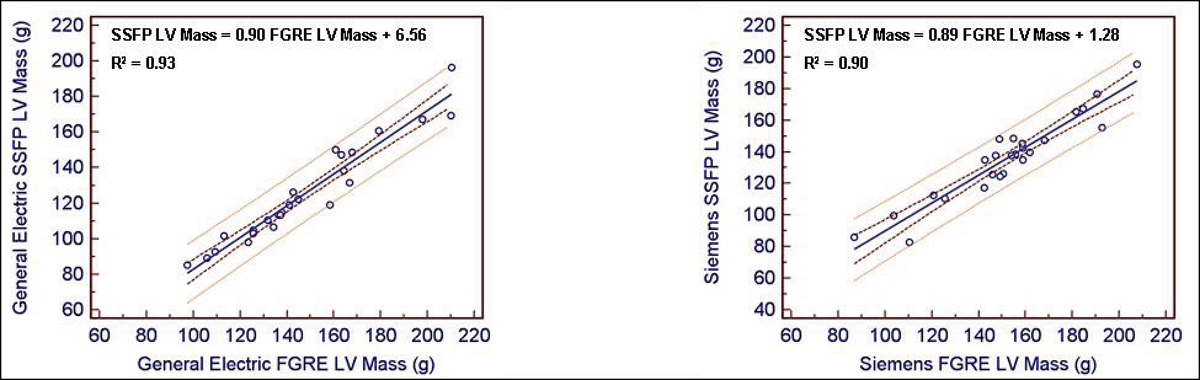
Figure 1

50 cardiac MRI cine studies (25 Siemens 1.5 T, 25 General Electric 1.5 T) were acquired using both SSFP and FGRE pulse sequences in the same study subject. Cardiac MRI analysis was performed using QMASS software to determine parameters of cardiac function including left ventricular (LV) mass, end diastolic volume (EDV), end systolic volume (ESV), ejection fraction (EF), stroke volume (SV), and cardiac output (CO). MRI analysis was first performed by a single trained reader. Interobserver variability was then assessed compared to a second reader. Results were assessed by regression and Bland-Altman analyses (Figure 1).

## Results

Mean percentage differences between SSFP and FGRE values for LV mass, EDV, and ESV were -10.5%, +17.7%, and +18.6% for Siemens scanners and -14.9%, +12.8%, and +15.0% for General Electric scanners, respectively (p < 0.0001 for all values). The relationship between SSFP and FGRE values using General Electric scanners were linear and highly correlated (p < 0.001) for LV mass, EDV, and ESV (R^2^ of 0.93, 0.87, and 0.91, respectively). For Siemens scanners, the relationship between SSFP and FGRE was highly correlated (p < 0.001) for LV mass (R^2^ = 0.90) and moderately correlated (p < 0.001) for EDV (R^2^ = 0.67) and ESV (R^2^ = 0.61). The slope (intercept) for EF, SV, and CO were 0.67 (22.4%), 0.73 (29.5 ml), and 0.61 (2.25 l/min) for Siemens scanners and 0.81 (11.1%), 0.90 (13.7 ml), and 0.95 (0.63 l/min) for General Electric scanners, respectively. Inter-observer mean differences (± 2 SD) for LV mass, EDV, and ESV using Siemens scanners were -2.1 g (-21.9–17.7 g), -16.5 ml (-35.4–2.4 ml), and -12.1 ml (-32.8–8.5 ml) for SSFP measures and -12.1 g (-48.1–24.0 ml), -18.7 ml (-37.7–0.3 ml), and -9.7 ml (-21.0–1.5 ml) for FGRE measures, respectively.

## Conclusion

Vendor-specific differences exist when comparing the relationships between SSFP and FGRE cine MRI for parameters of cardiac function.

